# Importance of powder diffraction raw data archival in a curated database for materials science applications

**DOI:** 10.1107/S2052520624006607

**Published:** 2024-08-26

**Authors:** Soorya Kabekkodu, Thomas Blanton

**Affiliations:** a International Centre for Diffraction Data 12 Campus Blvd. Newtown Square Pennsylvania19073 USA; Wilfrid Laurier University, Waterloo, Ontario, Canada

**Keywords:** powder diffraction, raw data, phase identification, Powder Diffraction File

## Abstract

In recent years, there is a significant interest from the crystallographic and materials science communities to have access to raw diffraction data. The effort in archiving raw data for access by the user community is spearheaded by the International Union of Crystallography (IUCr) Committee on Data. In materials science, where powder diffraction is extensively used, the challenge in archiving raw data is different to that from single crystal data, owing to the very nature of the contributions involved. Powder diffraction (X-ray or neutron) data consist of contributions from the material under study as well as instrument specific parameters. Having raw powder diffraction data can be essential in cases of analysing materials with poor crystallinity, disorder, micro structure (size/strain) *etc*. Here, the initiative and progress made by the International Centre for Diffraction Data (ICDD^R^) in archiving powder X-ray diffraction raw data in the Powder Diffraction File^TM^ (PDF^R^) database is outlined. The upcoming 2025 release of the PDF-5+ database will have more than 20 800 raw powder diffraction patterns that are available for reference.

## Abstract 2

Conventional *d*-spacing and relative intensity data found in the Powder Diffraction File database are enhanced with the inclusion of raw diffraction data, especially in characterizing samples with poor crystallinity, disorder, and interesting microstructure.

## 1. Introduction

The powder X-ray diffraction (PXRD) method, first demonstrated in 1916 by Debye and Scherrer, has remained a vital technique in materials characterization for over a century and is an indispensable analytical tool. One advantage of the powder X-ray diffraction (PXRD) method is that it often serves as a fingerprint of the solid-state materials under study. The powder diffraction method is a vital characterization tool for phases where growing a single crystal is difficult (or not attainable in practice) or the phase of interest is a part of a mixture.

In earlier decades the PXRD technique’s applications were limited to qualitative and semi-quantitative phase analysis and macroscopic stress measurement (Dinnebier & Billinge, 2008 ▸). The principal limitation of PXRD was the very nature of powder diffraction where three-dimensional reciprocal space is usually projected on a one-dimensional angular (2θ) axis resulting in severe peak overlaps. The advances made in the instrumentation and methodologies in the later part of the 20th century have opened up a wide variety of applications, from complex materials identification, quantification, and structure elucidation to micro structure and texture analysis (Gilmore *et al.*, 2019 ▸). In recent years, the advent of automated diffractometers (equipped with special environment sample cells) and two-dimensional (2D) detectors have made *in-situ* monitoring of complex chemical reactions and non-ambient parametric studies possible (Gilmore *et al.*, 2019 ▸).

**Figure 1:**
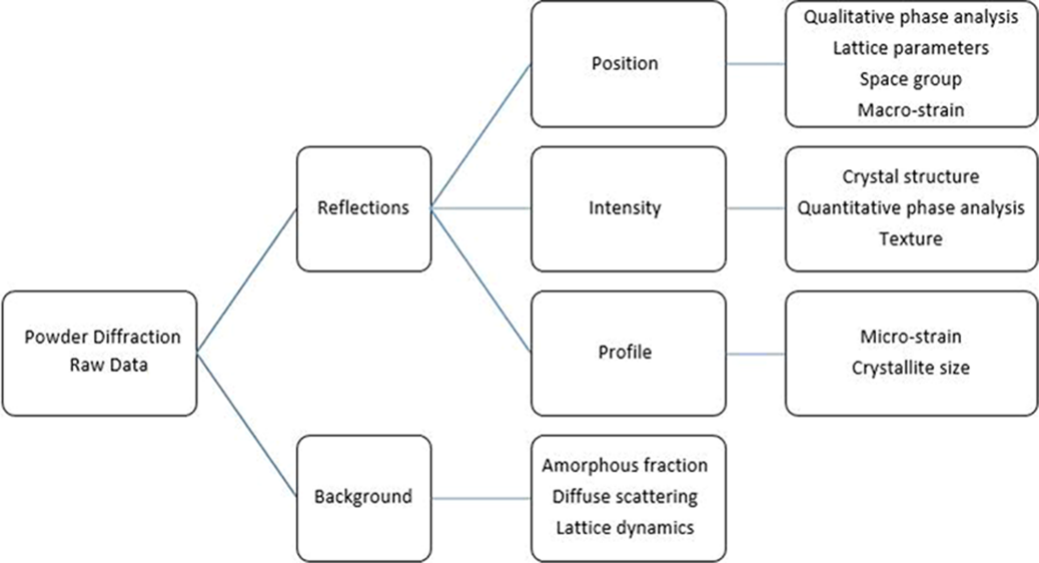
Information content of a powder diffraction pattern (adapted from Dinnebier & Billinge, 2008 ▸).

In the powder diffraction method, ‘powder’ refers to a large number of tiny crystallites randomly oriented. Sometimes PXRD is referred to as the polycrystalline diffraction method. A theoretical estimate is that at least 50 000 crystallites in the X-ray illuminated specimen volume are necessary to obtain a random powder diffraction pattern (Smith, 2001 ▸; Whitfield *et al.*, 2019 ▸). However, in practice, many samples are not powders, and materials under study can range from solid blocks of a metal to polymer gels. Unlike the single-crystal method, powder diffraction provides a wealth of information on the bulk material as shown in Fig. 1 ▸.

## 2. The Powder Diffraction File

The Powder Diffraction File (PDF), managed and maintained by the International Centre for Diffraction Data (ICDD, www.icdd.com), is a powerful database for materials characterization that has been used extensively by the scientific community for more than eight decades. The ICDD is a non-profit scientific organization dedicated to collecting, editing, publishing and distributing powder diffraction data to identify materials. Starting with approximately 1000 PXRD data entries on printed cards in 1941, the database has grown to contain over one million unique material data sets. The history, growth and development of the Powder Diffraction File has been summarized in various publications (Messick, 2012 ▸; Kaduk, 2019 ▸; Kabekkodu, Dosen & Blanton, 2024 ▸ and references therein). The Powder Diffraction File in Relational Database (RDB) format contains extensive chemical, physical, bibliographic and crystallographic data including atomic coordinates enabling characterization and computational analysis. In this paper we describe the ICDD’s implementation of archiving raw data and its availability for use by analysts in materials characterization.

### 2.1. Archiving raw powder diffraction data

The interest in access to raw data and its publication for the diffraction user community is beneficial to the fields of crystallography and materials science, and the IUCr has been promoting this initiative, as has the ICDD for many years. The review papers aptly titled ‘Science in the Data’ (Helliwell *et al.*, 2017 ▸, and reference therein) and ‘Raw diffraction data and reproducibility’ (Kroon-Batenburg *et al.*, 2024 ▸) elegantly encapsulate the need for raw data in crystallography. These papers detail archiving diffraction data in chemical crystallography which is predominantly single crystal. The challenges in archiving powder diffraction raw data will be discussed in later sections.

**Figure 2:**
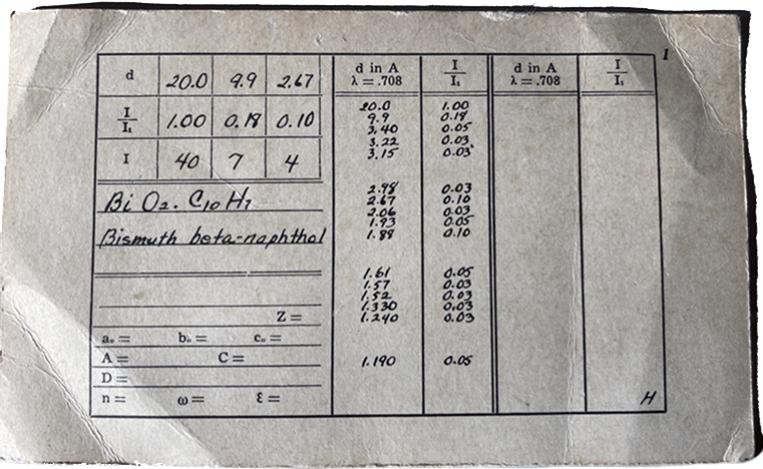
The first PDF card showing handwritten experimentally observed *d* spacings and relative intensities.

**Figure 3:**
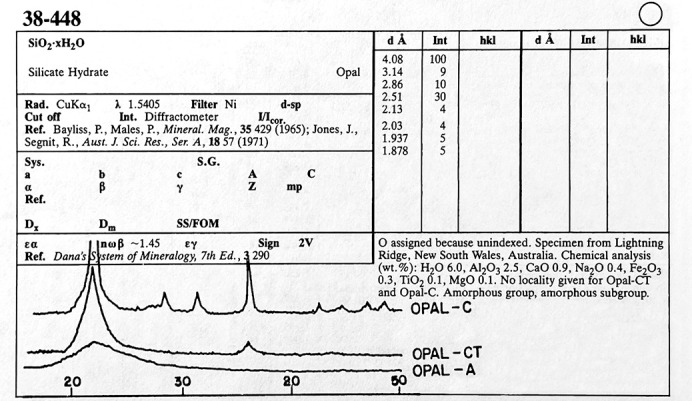
PDF card of the mineral opal with a PXRD image to facilitate phase identification.

Set 1 of the PDF published in 1941, on a 3 in × 5 in paper card (Fig. 2 ▸) was a listing of experimentally observed interplanar spacings (*d* spacings) and relative intensities (*I*/*I*_0_) characteristic of the compound. The ICDD first incorporated amorphous and poorly crystalline patterns in Set 38 (published in 1979), represented as a drawing of an amorphous silicate. The conventional listing of *d*-spacing and relative intensities was no longer sufficient to detail the diffraction pattern aspects and for identifying these types of poorly crystalline phases. The powder diffraction data (whole pattern) was published as an image (Fig. 3 ▸) back then due to the limitation of an analog book format.

**Figure 4:**
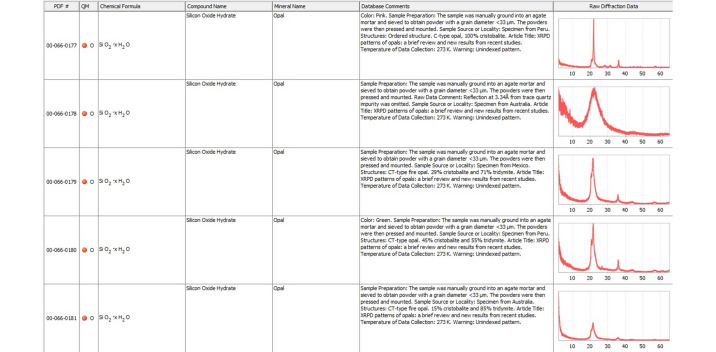
Powder diffraction raw data of different opal minerals in PDF-5+ 2024.

This was the genesis of the ICDD’s effort to archive raw powder diffraction data. In 1984 supported by the ICDD, a round robin study conducted on systematic errors found access to powder X-ray diffraction raw data was helpful in establishing the early guidelines for the deposition of raw data. (Schreiner & Fawcett, 1984 ▸). An outcome of this round robin was that the ICDD established a distinctive program called Grant-in-Aid (GiA) to acquire high-quality powder diffraction data of targeted materials. In the late 1980s, the ICDD emphasized GiA participants to deposit their raw powder diffraction data. The deposition of powder diffraction raw data is mandatory in the GiA program today. All new grantees are expected to submit powder diffraction raw data collected on a NIST (National Institute for Standards and Technology, www.nist.gov) Standard Reference Material (SRM) in order to evaluate the diffraction data quality prior to the grant approval. More detailed information about this grant is available on the ICDD website (https://www.icdd.com/grant-in-aid/). Other sources of raw data found in the PDF are from published literature, author contributed and private communications. Active involvement of the scientific community is crucial in the development and growth of any scientific database. The ICDD has been encouraging scientists to deposit their powder diffraction data using a web portal (https://www.icdd.com/data-submission/). Compared to the earlier example (Fig. 3 ▸) of the mineral opal, Fig. 4 ▸ shows raw PXRD data of different opals in PDF-5+ Release 2024 illustrating the progress made in archiving and presentation of raw PXRD data.

### 2.2. Data curation

All of the data published in the Powder Diffraction File goes through a multi-tier editorial process. Each entry in the PDF has an editorially assigned quality mark (Kabekkodu, Dosen & Blanton, 2024 ▸). An editorial comment will describe the reason if an entry does not meet the top-quality mark. The editorial processes of the ICDD’s quality management system are unique in that they are ISO 9001:2015 certified.

The challenges in archiving powder diffraction raw data are manifold due to phase impurities, data collection strategies, diffractometer geometry, sample preparation, particle statistics, systematic errors, and preferred orientation. These topics are covered in depth in *International Tables for Crystallography Volume H: Powder Diffraction* (2019 ▸) and in many reference books (Klug & Alexander, 1954 ▸; Jenkins & Snyder, 1996 ▸; Pecharsky & Zavalij, 2009 ▸; Dinnebier & Billinge, 2008 ▸). In our experience in reviewing submitted raw data about 30% of the raw data has issues that require contacting the authors. As an example, published raw data of sodium alginate [Fig. 6a in Chhatbar *et al.* (2009 ▸)] shows several sharp crystalline peaks, which is not characteristic of a typical polysaccharide biopolymer. Chhatbar *et al.* (2009 ▸) do mention washing sodium alginate with 0.25 *M* H_2_SO_4_, and further review of the data confirmed the highly crystalline phase to be sodium sulfate (Na_2_SO_4_, thenardite, PDF# 01-070-1541) overlapped with residual sodium alginate.

### 2.3. Importance of metadata

The FAIR (findability, accessibility, interoperability, and reusability) data principles (Wilkinson *et al.*, 2016 ▸) have been discussed widely in the scientific data archival effort in recent years. The role of metadata in terms of reproducibility of raw diffraction data were detailed in a recent publication by Kroon-Batenburg *et al*. (2024 ▸).

**Figure 5:**
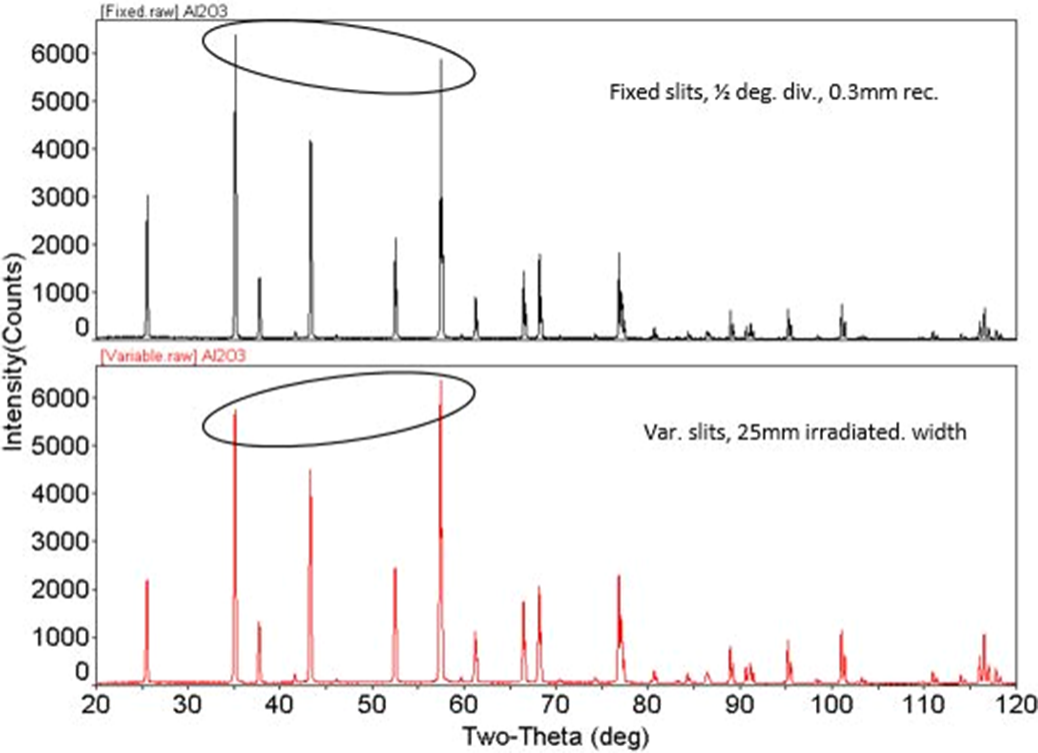
Comparison of NIST SRM 1976 plate PXRD patterns fixed versus variable slits. Notice the difference in the relative intensities (marked in the figure).

As discussed in the previous section, there are many factors that contribute to the powder diffraction raw data. It is extremely important to capture this information (where available) as metadata during the raw data archival. If one wishes to perform further analysis, for example, Rietveld refinement (Rietveld, 1967 ▸, 1969 ▸) then an instrument parameter file is required. A very basic correction for the sample displacement (one of the most commonly seen problems in Bragg–Brentano geometry) requires knowledge of the goniometer radius. Fig. 5 ▸ is a simple example demonstrating the influence of fixed versus variable slits on a diffractometer, affecting the normalized relative intensities of the PXRD pattern of NIST SRM 1976 (Al_2_O_3_). Many of the published PXRD raw data deposited as an XY ASCII file and archival of such raw data may not be useful without proper metadata in terms of its reusability and reproducibility. Much of the powder diffraction data is collected using home laboratory diffractometers, and the format varies with across instrument manufacturers.

**Figure 6:**
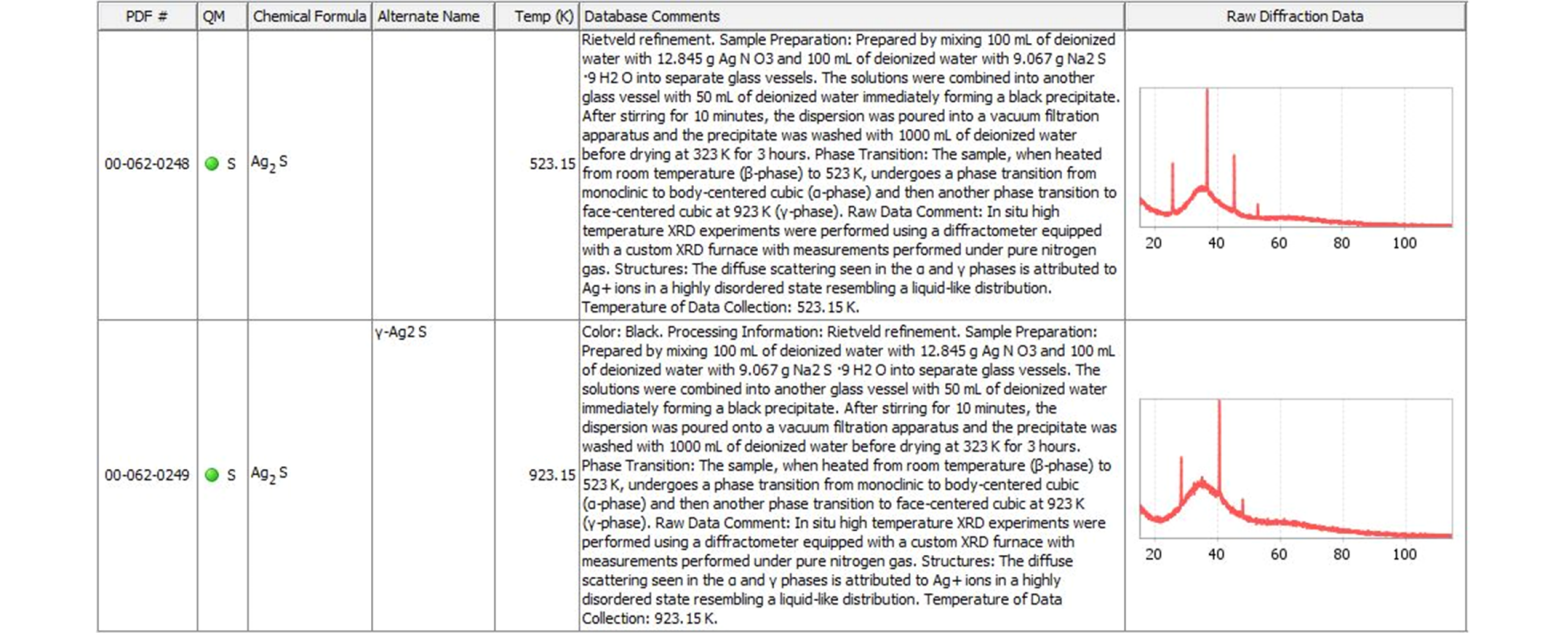
Metadata describing the diffuse scattering in α-Ag_2_S (at 523.15 K) and γ-Ag_2_S (at 923.15 K).

In some cases, the importance of metadata goes beyond the instrument configuration and data collection method adopted. Fig. 6 ▸ shows high-temperature polymorphs of silver sulfide, α-Ag_2_S (at 523.15 K) and γ-Ag_2_S (at 923.15 K). The authors (Blanton *et al.*, 2011 ▸) deposited metadata attributing the observed diffuse scattering to the highly disordered state of Ag^+^ ions at high temperature resembling a liquid-like distribution. Such a description is crucial in using these types of raw data. The ICDD’s online data submission tool enables metadata incorporation by prompting the authors to include them.

### 2.4. Raw data reusability

**Figure 7:**
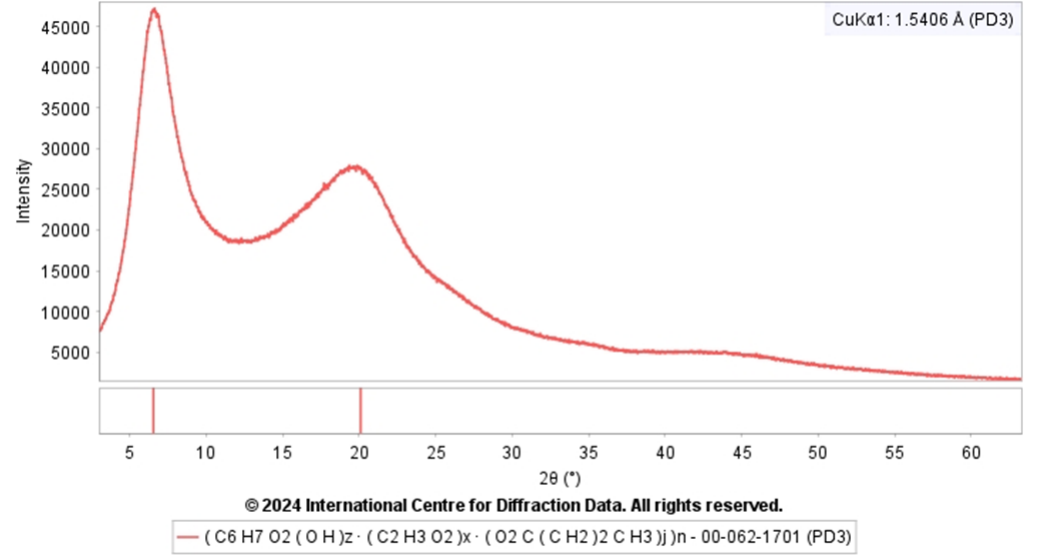
PXRD raw data of cellulose acetate butyrate (PDF# 00-062-1701).

An example of raw data reusability is the determination of the crystal structure of trandolapril (Reid *et al.*, 2016 ▸) using deposited raw data from the ICDD PDF. Most of the reusability of powder diffraction raw data is in the materials characterization where such data is a necessity, especially in characterizing poorly crystalline (clays, for example) or amorphous materials. It is evident from Fig. 7 ▸ that having raw data is essential to carry out phase identification in the case of poorly crystalline or amorphous patterns as they cannot be represented satisfactorily as an interplanar spacing (*d*) and relative intensity (*I*) list due to poorly resolved broad peaks. Whole raw data matching using a similarity index (Hofmann & Kuleshova, 2005 ▸) is one of the best methods to perform a search/match for poorly ordered phases. There are several examples where raw data is essential in characterizing pharmaceutical samples (Fawcett *et al.*, 2019 ▸), non-crystalline materials (Fawcett *et al.*, 2020*a* ▸,*b* ▸) and polymers (Gates *et al.*, 2014 ▸).

**Figure 8:**
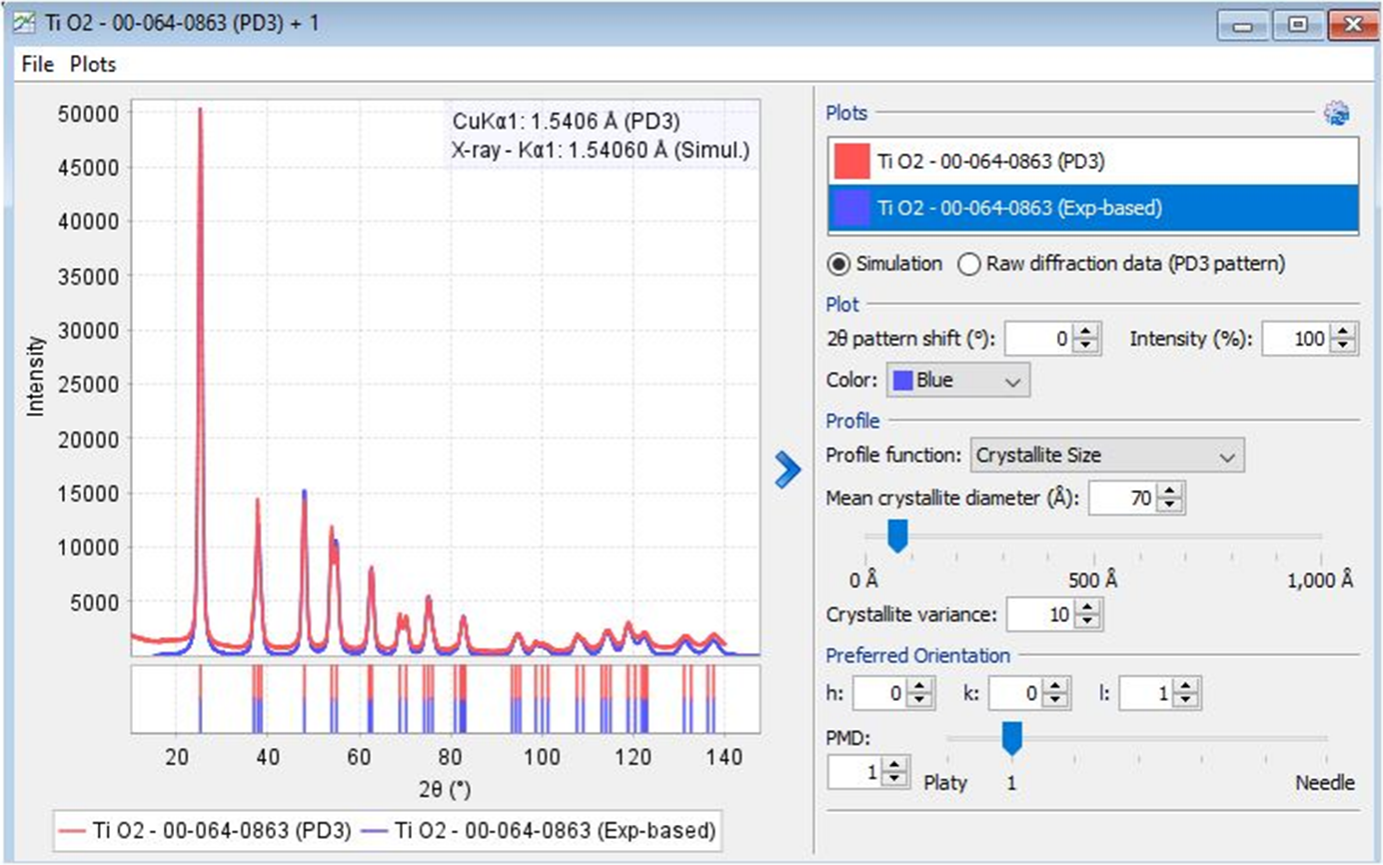
Estimation of the mean crystallite diameter of TiO_2_ (anatase, nano). Simulation (blue) with 70 Å mean crystallite diameter matches satisfactorily with the raw data (red).

Raw powder diffraction patterns of nanocrystalline powders are crucial in describing the microstructural features such as crystallite size. PDF-5+ software allows users to overlay simulated PXRD patterns by varying the crystallite size (Scardi *et al.*, 2006 ▸) and is beneficial in estimating the mean crystallite diameter of nanocrystalline powders. This feature is applicable to both user imported as well as archived raw data in the database. As an example, the estimation of the crystallite size of nano anatase (PDF# 00-064-0863) is shown in Fig. 8 ▸.

## 3. Future

Raw data that is currently archived by the ICDD is in one-dimension (1D) format primarily due to the nature of data collection using traditional powder diffractometers. In the future, the ICDD will encourage 2D raw data submission which will be beneficial in characterizing materials with texture and preferred orientation. Archiving time-of-flight neutron powder diffraction patterns is currently in development.

## 4. Conclusion

The availability of powder diffraction raw data in a well curated database plays a pivotal role in any successful material characterization or data driven study. The upcoming Release 2025 of the Powder Diffraction File will have more than 20 800 powder diffraction raw data entries. The exponential growth and interest in data-driven research based on machine learning and artificial intelligence make it critical to have a database with reliable data curation. One of the main problems in archiving powder diffraction raw data is the lack of a common format that would encapsulate parameters that have a strong influence on the observed powder diffraction pattern. Scientific journals encouraging authors to submit powder diffraction raw data as powder CIF (https://www.iucr.org/resources/cif/dictionaries/cif_pd) along with required metadata will be beneficial for the materials science community.
